# What Homophones Say about Words

**DOI:** 10.1371/journal.pone.0162176

**Published:** 2016-09-01

**Authors:** Isabelle Dautriche, Emmanuel Chemla

**Affiliations:** Laboratoire de Sciences Cognitives et Psycholinguistique, (DEC-ENS/EHESS/CNRS), Paris, France; University of Akron, UNITED STATES

## Abstract

The number of potential meanings for a new word is astronomic. To make the word-learning problem tractable, one must restrict the hypothesis space. To do so, current word learning accounts often incorporate constraints about cognition or about the mature lexicon directly in the learning device. We are concerned with the convexity constraint, which holds that concepts (privileged sets of entities that we think of as “coherent”) do not have gaps (if A and B belong to a concept, so does any entity “between” A and B). To leverage from it a linguistic constraint, learning algorithms have percolated this constraint from concepts, to word forms: some algorithms rely on the possibility that word forms are associated with convex sets of objects. Yet this does have to be the case: homophones are word forms associated with two separate words and meanings. Two sets of experiments show that when evidence suggests that a novel label is associated with a disjoint (non-convex) set of objects, either a) because there is a gap in conceptual space between the learning exemplars for a given word or b) because of the intervention of other lexical items in that gap, adults prefer to postulate homophony, where a single word form is associated with two separate words and meanings, rather than inferring that the word could have a disjunctive, discontinuous meaning. These results about homophony must be integrated to current word learning algorithms. We conclude by arguing for a weaker specialization of word learning algorithms, which too often could miss important constraints by focusing on a restricted empirical basis (e.g., non-homophonous content words).

## Introduction

Learning the word “cat” implies associating the sequence of sounds /kaet/ to the set of all cats and only cats. Quite generally one description of the meaning of a content word is its “extension”, i.e. the set of all entities to which that word refers (an idea discussed in detail in the tradition of formal semantics at least since [1]). But language learners need to infer the extension of a word based on a set of exemplars that surely do not exhaust that extension. The underlying inference problem would be unsolvable without prior knowledge, most notably some that could constrain the hypothesis space, which is the set of potential meanings for words (e.g., [2]–[5]; and [6] for a formal proof).

One way in which learners may reduce their hypothesis space is by privileging some meanings over others. For instance, toddlers and preschoolers prefer to extend a novel word (e.g., assume “blicket” is first associated with a dog) to an object of the same kind (e.g., a cat) rather than to an object of a different kind (e.g., a bone) (e.g., [7], [8]; see also the “shape bias”, showing that infants extend a label on the basis of the shape, [9]).

This follows if learners assume that those concepts that have words associated with them, are *convex* (e.g., [10]). A concept is convex if its members form a group that share a common set of properties that holds them to be contiguous in conceptual space (e.g., [10], see also [11] for the idea of “conceptual coherence”). For instance, the category dog or bone is not a possible concept because it does not form a coherent class of objects. Thus, if concepts are expected to be convex and words label concepts, learners may more readily extend the extension of a word (e.g., “blicket” designing a dog) to neighboring objects in conceptual space (cat) rather than to more distant objects (bone).

Thus, current experimental results provide evidence that a convexity constraint guides learners' inferences about word meanings: if A and B can be labeled using the sound /bliket/, then all objects falling “in between” A and B in conceptual space can also be labeled with the sound /bliket/. But these experiments do not distinguish between words and word forms, hence it is unclear whether this constraint applies at the level of the words or at the level of the word forms.

Yet words and word forms can be dissociated. A homophone is a phonological form associated arbitrarily with *several* meanings (contrary to polysemy, see e.g., Bréal, 1904), which together form a discontinuous set in conceptual space. For instance, the English word form “bat” applies both to the convex concept of animal bats and to the convex concept of baseball bats, but, regardless of how the conceptual space is constructed, not all intervening objects sharing a common property of animal bats and baseball bats count as bats.

Although the domain of application of the convexity constraint, words or word forms, is rarely specified explicitly, all current models of word learning (e.g., [12], [13], [14]) practically implement a convexity constraint at the level of word forms. It is a virtue of these word learning models that they work at the level of word forms, because this is the visible layer of the input, learners hear word forms, not words. But note that as a consequence of this implementation, these accounts mechanically predict that when encountering a word form that applies to animal bats and baseball bats, English learners should conclude that *bat* applies to any intervening object, as would words that apply very broadly, such as “thing” or “stuff”. The very existence of homophony in human languages thus shows that learners do not adhere blindly to a convexity constraint on word forms. In sum, learners make inferences about the meaning of words, based on the occurrence of some word forms across different situations, We will show how learners rely on the (hidden) *word* level of representation to master a lexicon and how they may capitalize on a proper convexity constraint at this level to learn homophones.

Concretely, our point of departure will be work by Xu and Tenenbaum (2007) [12]. One advantage of their study is that it implements the convexity constraint on word forms in a predictive model, but it also provides the means to test it in a non-circular way. To do so, they first gathered similarity judgments between pairs of objects, and inferred a tree-structure over the whole set. This tree structure represents the taxonomy between the objects: different dogs are close together and form a subtree, mammals form a (bigger) subtree, etc. Such a hypothesis space reflects the taxonomic assumption [5] that requires words to label the nodes of a tree-structured hierarchy of natural concepts, in line with developmental data (e.g., [4], [5], [7], [8]. Crucially, Xu and Tenenbaum (2007) [12] used this structured conceptual space to test a model of word learning according to which the extension inferred for a given word label should be a set of objects with no gap in conceptual space and which minimally includes all exemplars. Thus, *intervening* objects, i.e., objects that are *in between* two learning exemplars, are defined as all objects in the smallest subtree that includes both exemplars (their convex hull). Accordingly, the authors demonstrate that, when exposed to a set of learning exemplars, participants extend the exemplars’ label to all intervening objects belonging to the smallest subtree that contains all these exemplars. For example, when presented with three Dalmatians as exemplars for a new word “fep”, adults readily extend “fep” to the set of all Dalmatians; would they be presented with a Dalmatian, a Labrador and a German-shepherd for the word “fep”, they would extend the label to the set of all dogs. In other words, participants pick the smallest generalization that satisfies the convexity constraint on word forms.

The present study explores the situations that lead language learners to postulate homophony for a new word using the word learning paradigm used by Xu and Tenenbaum (2007) [12]. In Experiment 1, we manipulate two factors that should invite learners to favor a homophone interpretation of a novel label:

1. The *size of the gap*, in conceptual space, that separates different learning exemplars of a given word. To learn a homophone, language learners are exposed to a discrete set of learning exemplars. For instance, for the word *bat*, they would observe several animal-bats and several baseball bats. However if the underlying true concept were the broad category that encompasses animal-bats, baseball-bats and all intervening objects (e.g., “thing”), then presumably learners would not observe exemplars confined to two corners of this set. Rather, they would observe a *set* of learning exemplars randomly (uniformly) sampled from the broad category. Observing exemplars clustered at two distant positions in the hypothesis space, i.e., observing a large gap between the exemplars may boost the likelihood that the exemplars are sampled from two independent categories, favoring a homophone interpretation.
2. *The intervention of other lexical items in that gap*. Evidence for homophony may also come from other words in the lexicon. There has been much evidence that words and their underlying concepts mutually constrain each other. For instance, language learners assume that words do not overlap in meaning (the “mutual exclusivity effect”; e.g., [15]). Having evidence that an additional label point towards an intervening region of the conceptual space (e.g., between animal-bat and baseball bats) may help learners discover more subtle configurations about how words map onto meanings.

Our results show that participants refrain from associating a label to a broad concept encompassing all the exemplars. Yet it does not entail that learners postulate homophony in these cases: Learners could have accepted that a word map onto a discontinuous concept (e.g., dog or bone) therefore violating concept convexity. We address this question more directly in Experiment 2. All in all, our results suggest that the effects documented in Experiment 1 are the footprints of homophony: Learners prefer to associate a single word form to several words and associated convex concepts, thus preserving concept convexity at the expense of word form convexity. This shows that current accounts of word learning face new challenges when incorporating homophony into the picture and that homophony can reveal (some of) the existing constraints learners deploy while learning words.

## Experiment 1: Gap in Conceptual Space and Overall Structure of the Lexicon

We used a word learning *paradigm à* la Xu and Tenenbaum (2007) [12]: participants were exposed to a new label through a couple of learning exemplars and asked whether the label should be extended to test items. We introduced a) a large gap in conceptual space between learning exemplars b) an intervening exemplar with a different label in that gap. We predicted that these two manipulations would lead to a breaking point after which participants would violate a convexity constraint on word forms, i.e., exclude items in the gap from the extension of the label.

### Method

#### Ethic Statement

All research was approved by the Comité d'Ethique de la Recherche en Santé (2013/46). Following the committee's recommendations, prior to accepting to participate in the online studies, participants were presented with the informed consent document and instructions stating that by clicking “Agree” they indicated their consent to participate in the study.

#### Participants

One hundred and five adults were recruited through Amazon’s Mechanical Turk (45 females; M = 33 years; 102 native speakers of English) and compensated $0.50 for their participation. We excluded participants for lack of engagement in the task (criterion: participants who selected no test item in more than 50% of the “attractive” trials, in which at least 3 items should have been selected, see below; *n* = 0 in Experiment 1A, *n =* 16 in Experiment 1B) and participated in both versions of the experiment or in a previous pilot version (*n* = 3 and 5). This resulted in 41 participants in Experiment 1A and 40 participants in Experiment 1B. Data collection was stopped when each of the experiment had at least 40 participants. The number of participants was established before data collection began.

#### Procedure and display

**Fig 1:**
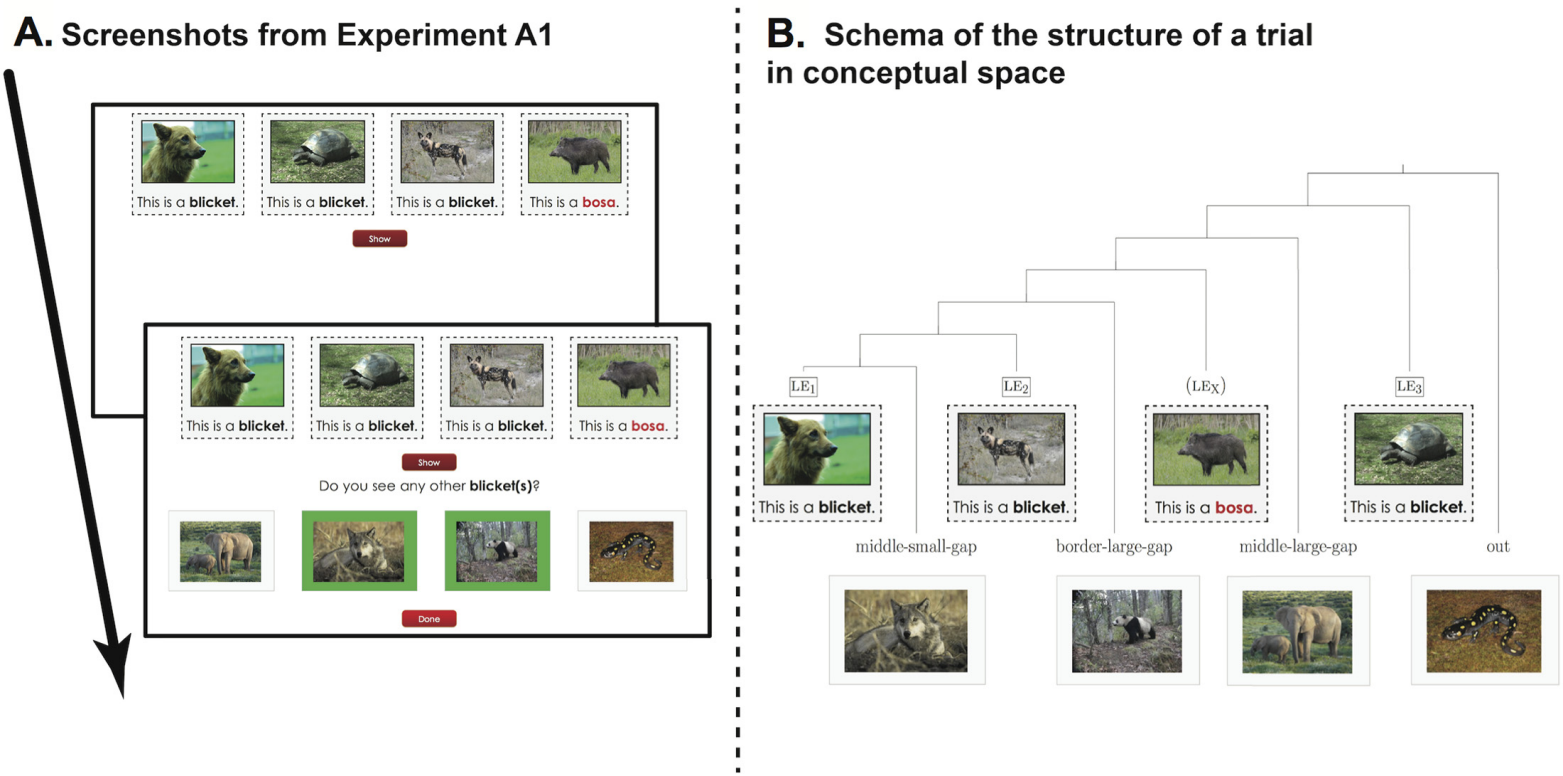
**A) Screenshots from Experiment 1A.** Participants first see the 3 learning exemplars for the word “blicket” and one optional learning exemplar for the word “bosa”. After pressing the “show” button they then see the test pictures and are asked to find the other blickets. Once the pictures are selected (green frame), participants submit their answers by pressing the “done” button. **B) Schema of the structure of a trial in conceptual space.** The first row of pictures corresponds to the learning exemplars (le1, le2, le3, leX) and the second row to the test items. The intervening item leX appeared only in half of the test trials (hence the parentheses).

Participants were tested online. They were instructed that they would be exposed to words from an alien language and would have to select images that correspond to those words. In the instructions, participants were shown an example of a trial with pictures and a label that would not appear during the test. In each trial, participants first saw 3 or 4 learning exemplars, presented as the combination of a picture and a sentence. The first three learning exemplars (referred to as le1, le2 and le3 below) were presented in random order and labeled with a novel word, e.g., *blicket*, via a prompt of the form “This is a blicket” underneath each of them. The fourth learning exemplar (leX below), if present, was labeled with another novel word highlighted in red, as in e.g., “This is a bosa” and was always the right-most exemplar. Once participants pressed a button “Show”, they would see a set of 4 pictures below the learning exemplars and be asked to select from these test items which one(s) could be labeled with the first novel word: *“Do you see any other blicket(s)*?*”* (see Fig 1A). They responded by clicking to select none, one or multiple test items. When a picture was selected, its frame became green. Participants could unselect their choice by clicking on it again. Once a response was validated, the set of selected pictures was recorded and the test continued to the next trial.

#### Conditions

Each participant saw 12 test trials and 10 filler trials.

*Test trials*. The structure of test trials is represented schematically in Fig 1A, the key factor is how the learning exemplars (le1, le2, le3 and optionally leX) were spread in conceptual space (here a tree-structure) and how the test items were distributed between them. As shown in Fig 1B, there were two gaps between the exemplars: one small gap between le1 and le2 and one much larger gap between le2 and le3. Test items were picked somewhere in the middle of the first small gap (*middle-small-gap*), of the large gap (*middle-large-gap*), in the large gap but close to the corresponding exemplars (*border-large-gap*) or out of all the exemplars altogether (*out*).

Six of the test trials, “Gap trials”, were designed solely to test the effect of the size of a gap between learning exemplars. They displayed three learning exemplars (le1, le2, le3) associated with a to-be-learned label. According to the convexity constraint on word forms, participants should select all test items in the minimal subtree containing all learning exemplars, but we expected that participants would be willing to violate this constraint and exclude *middle-large-gap* (or not as much as *middle-small-gap*).

Another 6 test trials, “Gap+Intervention trials”, had a fourth learning exemplar with a secondary label (the leX *bosa* exemplar in Fig 1). The convexity constraint on word forms applies to single lexical entries and is in principle blind to the rest of the lexicon, but we expected that participants would select the *middle-large-gap* test item less in these trials with an intervening label than in the test trials without this intervening label.

*Filler trials*. One filler trial was presented first so that participants could familiarize themselves with the task (with no particular indication of it however). Nine other fillers were randomly interspersed between the test trials. 6 “attractive” fillers were designed such that participants would select at least 3 of the 4 test pictures (3 of these filler trials contained three learning exemplars, all with the same label as in the Gap test trials, and 3 others included a fourth learning exemplar with a secondary label as in the Intervention test trials). 3 “repulsive” fillers implemented the opposite bias: participants were expected to select one or no test picture.

#### Materials

Our stimuli relied on a set of to-be-learned labels and taxonomically organized objects.

*Labels*. 28 phonotactically legal non-words of English were used for both experiments and were not repeated across trials.

**Fig 2:**
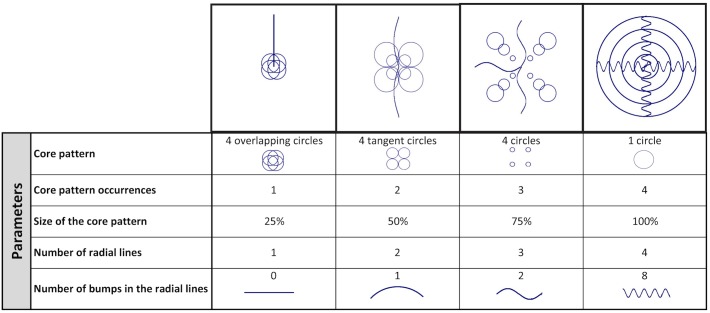
Stimuli of Experiment 1B. Examples of the artificial stimuli used in Experiment 1B, out of a set of 1024 possible unique combinations obtained from 5 parameters (core pattern, core pattern occurrences, size of the core pattern, number of radial lines, number of bumps in the radial lines) with 4 levels each.

*Objects in conceptual space*. We tested participants on two sets of objects organized into drastically different taxonomic hierarchies: natural objects, with a similarity measure based on phylogenetic trees (Experiment 1A) and artificial objects constructed in a parametric fashion, so that a similarity measure between these objects can be defined in a canonical way (Experiment 1B; Fig 2). Objects from this artificial taxonomy do not exist such that the actual lexicon of our participants cannot influence our experimental results.

One important difference with Xu and Tenenbaum’s [12] paradigm is that our conceptual space did not rely on subjective, experimentally-gathered similarity judgments, but rather on objective similarity measures: one based on the distance in the phylogenetic tree and the other based on the parameterization of the objects. Surely these measures are only a proxy for participants’ representation of the similarity relationships between the objects. Yet, any effect that can be detected from these imperfect objective measures will retrospectively validate that it is a good approximation of the underlying subjective measure. We describe the two sets of objects at the basis of Experiments 1A and 1B, their structure, and how our experimental conditions were obtained in each case in S1 Supplemental Material. The experimental material for both experiments is available at https://osf.io/u473e/?view_only=33576a1ac18746b08d7e3fcc96e10e9a

#### Presentation and trial generation

The order of the trials as well as the pairing between the labels and the set of learning exemplars was fully randomized and differed for each participant. All trials were generated automatically following the algorithmic constraints described in S1 Supplemental Material for each stimuli type.

#### Statistical analysis

In a mixed logit regression [16], we modeled the selection of a test item (coded as 0 or 1) for each experiment (natural or artificial stimuli). Both models included two categorical predictors with their interaction: Test Item (*middle-small-gap*, *border-large-gap*, *middle-large-gap*, *out*) and Trial Type (Gap vs. Gap+Intervention) as well as a random intercept and random slopes for both Test Item and Trial Type for participants. We coded our predictors such that selection of *middle-large-gap* for Gap trials served as a baseline (unless otherwise mentioned) against which we compared a) responses to the other test items, b) the responses to *middle-large-gap* in Gap+Intervention trials.

All analyses were conducted using the lme4 package [17] of R.

### Results

**Fig 3:**
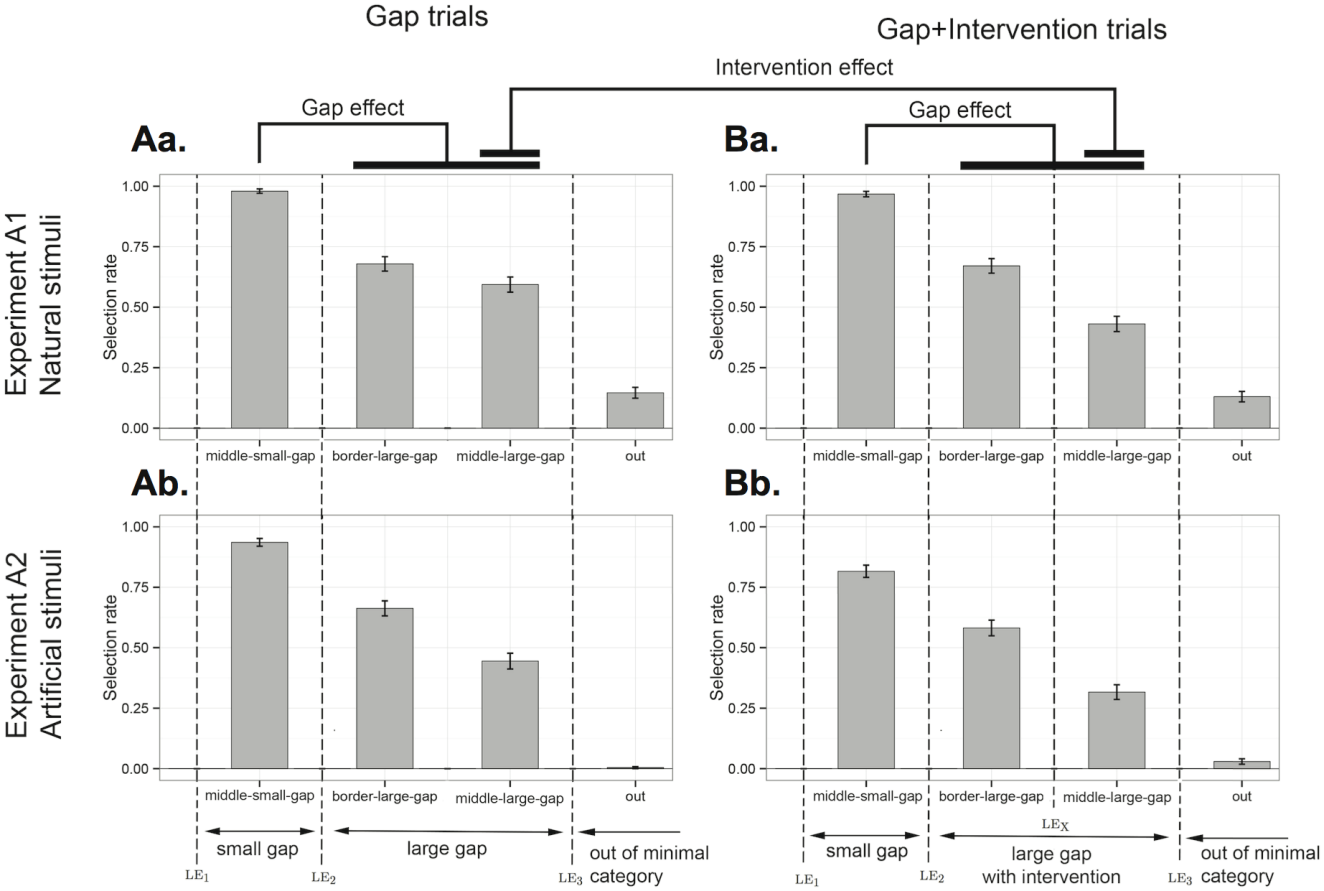
Results of Experiment 1. Proportion of choice of each test item averaged across Experiment 1A with natural objects (upper panel) and Experiment 1B with artificial objects (lower panel) for each trial type (Gap vs. Gap+Intervention trials). The x-axis follows (with some simplification) the structure in conceptual space: the position of the learning exemplars is indicated among the bars for the test items with the dashed lines. Error bars indicate standard errors of the mean.

Fig 3 reports the average proportion of selection of each test item by Trial Type (Gap vs. Gap+Intervention trials) and Experiment (1A or 1B).

For Gap trials (Fig 3Aa and 3Ab), we replicate the minimal category effect seen in previous results (i.e., [12]) showing that participants are more likely to select a test item belonging to the category which is minimally consistent with the exemplars (*middle-small-gap*, *border-large-gap*, *middle-large-gap*) than a test item outside of this category (*out*), both for Experiment 1A (β = -3.75, *z* = -12.20, *p* < .001) and Experiment 1B (β = -5.17, *z* = -11.46, *p* < .001; We Helmert-coded the predictor Test Item to compare the choice of *out* to the choice of the rest of the test items as a group). Crucially, the size of the gap between learning exemplars modulated participants’ responses. That is, participants selected *middle-small-gap* items more than *middle-large-gap* items both in Experiment 1A (*M*_*middle-large-gap*_ *=* 0.59, *M*_*middle-small-gap*_ *=* 0.98; β = 3.72, *z* = 7.53, *p* < .001) and in Experiment 1B (*M*_*middle-large-gap*_ *=* 0.44, *M*_*middle-small-gap*_ *=* 0.94; β = 3.45, *z* = 10.38, *p* < .001). Participants were sensitive to the distribution of the learning exemplars with natural stimuli but also with unfamiliar stimuli. This latter case shows that familiarity with the categories (e.g., mammals, carnivores, animals) and possible existing labels for them cannot fully explain the results.

For Intervention trials (Fig 3Ba and 3Bb), we first replicate the effect described above: participants were sensitive to the size of the gap between the exemplars, that is, they selected *middle-small-gap more* than *middle-large-gap* in Experiments 1A (*M*_*middle-large-gap*_ *=* 0.43, *M*_*middle-small-gap*_ *=* 0.97; β = 3.95, *z* = 9.75, *p* < .001) and in Experiment 1B (*M*_*middle-large-gap*_ *=* 0.32; *M*_*middle-small-gap*_ *=* 0.82; β = 2.70, *z* = 10.32, *p* < .001). Crucially, we expected that the presence of an intervening item would increase participants’ violation of a convexity constraint on word forms.

Indeed, in Experiment 1A, participants selected *middle-large-gap* less in Gap+Intervention trials than in Gap trials (β = -0.72, *z* = -3.80, *p* < .001). Yet, the presence of an intervening lexical item did not affect the choice of any other test items (all *ps* > 0.4) leading to an interaction effect: the difference between the selection rate of *middle-small-gap* and *middle-large-gap* was greater in Gap+Intervention trials than in Gap trials (β = 0.68, *z* = 2.51, *p* < .01).

In Experiment 1B, participants similarly selected *middle-large-gap* less in Gap+Intervention trials than in Gap trials (β = -0.61, *z* = -2.40, *p* < .05). But we should pause and note that the same was true for *middle-small-gap* items (β = -1.40, *z* = -3.96, *p* < .001; here the intercept reflected selection of *middle-small-gap* in Gap trials). This was because the intervening exemplar leX was sometimes close to *middle-small-gap* (and even closer than it was to *middle-large-gap*), thus introducing an independent reason not to select *middle-small-gap* in these intervention trials.

Overall, we did observe that intervening labels block the extension of a word to the minimal category including all observed exemplars, even though this effect was polluted for artificial stimuli.

### Discussion

We highlighted two factors that disturb the association of a word form to the single category that minimally includes all its learning exemplars: a) the size of the gap between the exemplars; b) the presence of intervening lexical items. There may be three potential interpretations for these results:

1. Participants associated a label to two meanings that *each* satisfies concept convexity. That is, participants postulated homophony, a non-immediate way to bind labels and concepts. Note however that we did not test whether participants provide evidence that subjects generalized from the more distant trained exemplar, le3, a point that will be addressed in the next experiment.
2. Participants associated a label with a set covering entities from several *disjoint* concepts (e.g., as in dog or bone), breaking thus concept convexity, either because meaning discontinuity is acceptable or because the specific experimental task that we propose led them to do so.
3. Participants did not associate the new word with a meaning at all. Instead, they simply went by similarity of the test items to the learning exemplars: they selected more the objects close to the exemplars (*middle-small-gap*) than to the objects further away from them (*middle*-*large-gap*). The role of the intervening label may be harder to account for in this view, but one may imagine some strategic effect such that if an object is close to some irrelevant object X, it will decrease the tendency to say that this object belongs to a set that was not said to contain X.

Experiment 2 was designed to distinguish between these three interpretations.

## Experiment 2: Linguistic Manipulations

Homophones interact with linguistic constructions in a characteristic way. Zeugmas are the typical rhetorical device used to pun on the different senses of ambiguous words (e.g., [18], [19]) and have been extensively used as a test to distinguish words with an extension that covers a broad category from polysemous and ambiguous words (e.g., [18], [20]). Consider for instance “John and his driving license expired last Thursday” [18], where the verb “expire” has two distinct, but related, senses (i.e. “died” and “not valid anymore”). If the zeugmatic sentence is acceptable, it shows that the relevant word is polysemous or ambiguous (the two meanings are distinct) rather than vague (the boundary between meanings are indistinct).

Interestingly, zeugmas can be used to distinguish between a homophone, where a label applies to two convex concepts, and a word associated with a disjunctive meaning, where a label would apply to a disjoint concept. For instance, if “blicket” maps onto a disjunctive concept, such as dog or bone, it should be possible to use a plural sentence “these are two blickets” when pointing to a dog and a bone, while it would be zeugmatic to say “these are two bats”, pointing at one animal-bat and one baseball-bat. This is explained in a theory of homophones in which two words, with different meanings, share the same form: one cannot use a single phonological form to refer to both meanings at the same time. However, different *tokens* of the phonological form may pick out different meanings: it may therefore be more natural to say in a situation as above “This is a bat (pointing at the animal-bat), this is *also* a bat (pointing at the baseball-bat)”.

We will use these two constructions to test whether the effects we documented in the experiments above are the signatures of homophony. If participants postulated homophony, the *plural* zeugmatic construction, which is not compatible with homophony, should increase the tendency to form a single convex category encompassing all learning exemplars (as dictated by the convexity constraint over word forms in the absence of homophony), compared to the *also* construction. This would be evidence that participants did not postulate that a label could map onto a discontinuous concept and that our effects are not solely driven by similarity, since the similarity of the test items to the exemplars is held constant across the two linguistic constructions.

### Method

#### Participants

Ninety adults were recruited through Amazon Mechanical Turk (28 females; M = 30 years; 87 native speakers of English) and were compensated $0.50 for their participation. We excluded subjects who participated in both conditions of the experiment (*n* = 3). This resulted in 44 participants in the *also*-condition and 43 participants in the *plural-*condition. Data collection was stopped when each of the conditions had at least 40 participants. The number of participants was established before data collection began.

#### Procedure and display

Similar to Experiment 1, except that each trial now included 4 learning exemplars and 6 test items.

#### Conditions

Each participant saw 8 test trials and 16 filler trials.

**Fig 4:**
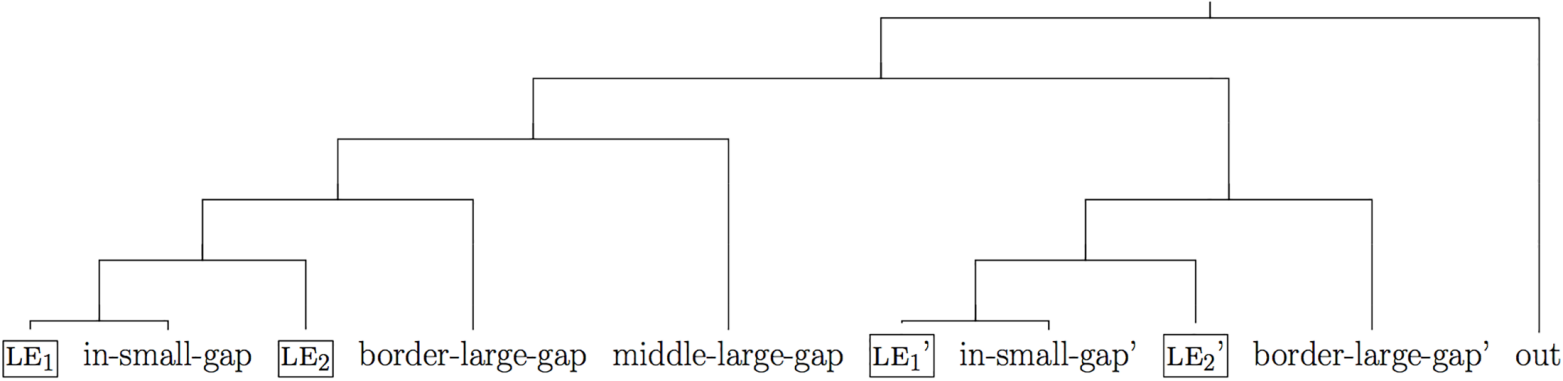
Schema of the tree-structure of the items used in a trial for Experiment 2. The boxed items correspond to the learning exemplars.

*Test trials*. As schematized in Fig 4, each test trial contained 4 learning exemplars (le1, le2 and le1’, le2’). We implemented symmetry in the distribution of learning exemplars such that there were two small gaps (between le1 and le2 and between le1’ and le2’) and one large gap (between the two pairs of exemplars). This distribution of exemplars in conceptual space may favor the construction of sharp boundaries over two disjoint categories (see also discussion of the “size principle” in the General Discussion). The position of the six test items is shown in Fig 4. Two test items were placed inside the small gaps (*middle-small-gap* and *middle-small-gap*’), two items just outside of the minimal subtrees S(le1,le2) and S(le1’,le2’) containing each pair of exemplars (*border-large-gap* and *border-large-gap*’), one item inside the large gap (*middle*-*large-gap*, either attached to S(le1,le2) or to S(le1’,le2’)) and one item outside of the minimal subtree containing all four learning exemplars (*out*).

**Fig 5:**
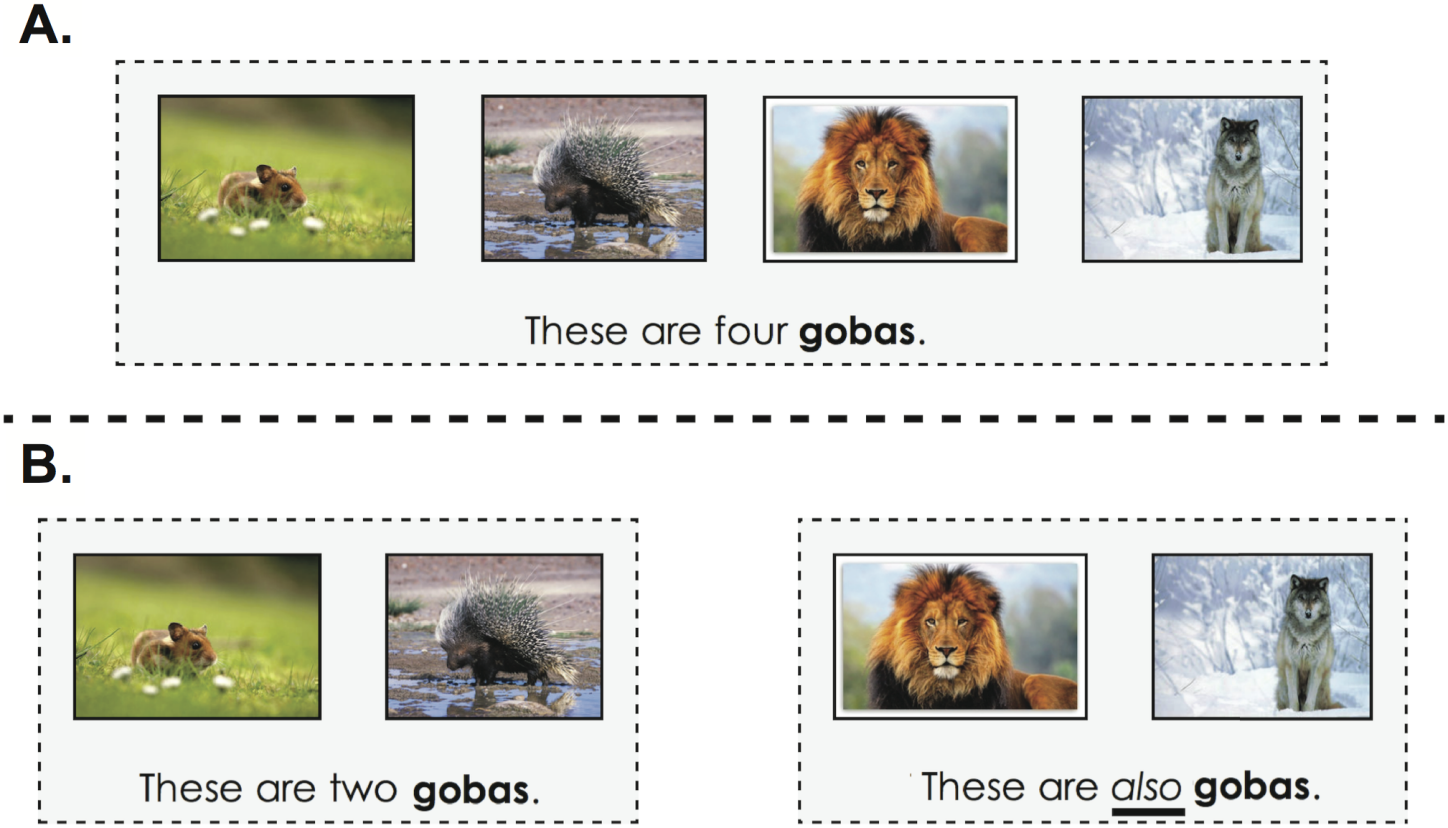
Example of a test trial in Experiment 2. Possible learning exemplars for a test trial as presented in 1) the plural-condition and 2) the also-condition.

The 8 test trials were created according to the schema in Fig 4, but their mode of presentation differed across the two conditions. In the *also*-condition, the four learning exemplars were presented in pairs: the left pair was labeled with a given word (e.g., “These are two blickets”) and the right pair with the same word using *also* (e.g., “These are *also* two blickets”). In the *plural*-condition, the four learning exemplars were ordered in pairs as in the *also*-condition but the four exemplars were grouped together in a gray frame and labeled at once via a plural sentence (e.g., “These are four blickets”; see Fig 5).

We expected that participants would select the test items *middle-large-gap* and *border-large-gap* more in the plural-condition than in the also-condition, because homophony is less of an option while using the plural construction.

*Filler trials*. 16 filler trials were interspersed, half of which were visually similar to the test trials of the plural-condition (Fig 5A) and the other half were visually similar to the test trials of also-condition (Fig 5B; but with a different label for the two pairs of objects and, of course, no *also* in the description).

#### Material

We used the same set of objects as in Experiment 1A and the same labels.

#### Presentation and trial generation

The experiment always started with 3 filler trials. All trials were generated pseudo-randomly following the constraints described in S1 Supplemental Material.

#### Statistical analysis

As before, we modeled the selection of a test item in a mixed logit model including two categorical predictors with their interaction: Test Item (*middle-small-gap*, *border-large-gap*, *middle-large-gap*, *out*) and Linguistic Condition (Plural vs. Also) as well as a random intercept and a random slope for Test Item on participants. The selection of *middle-large-gap* in the plural-condition served as a baseline (unless otherwise mentioned).

### Results

**Fig 6:**
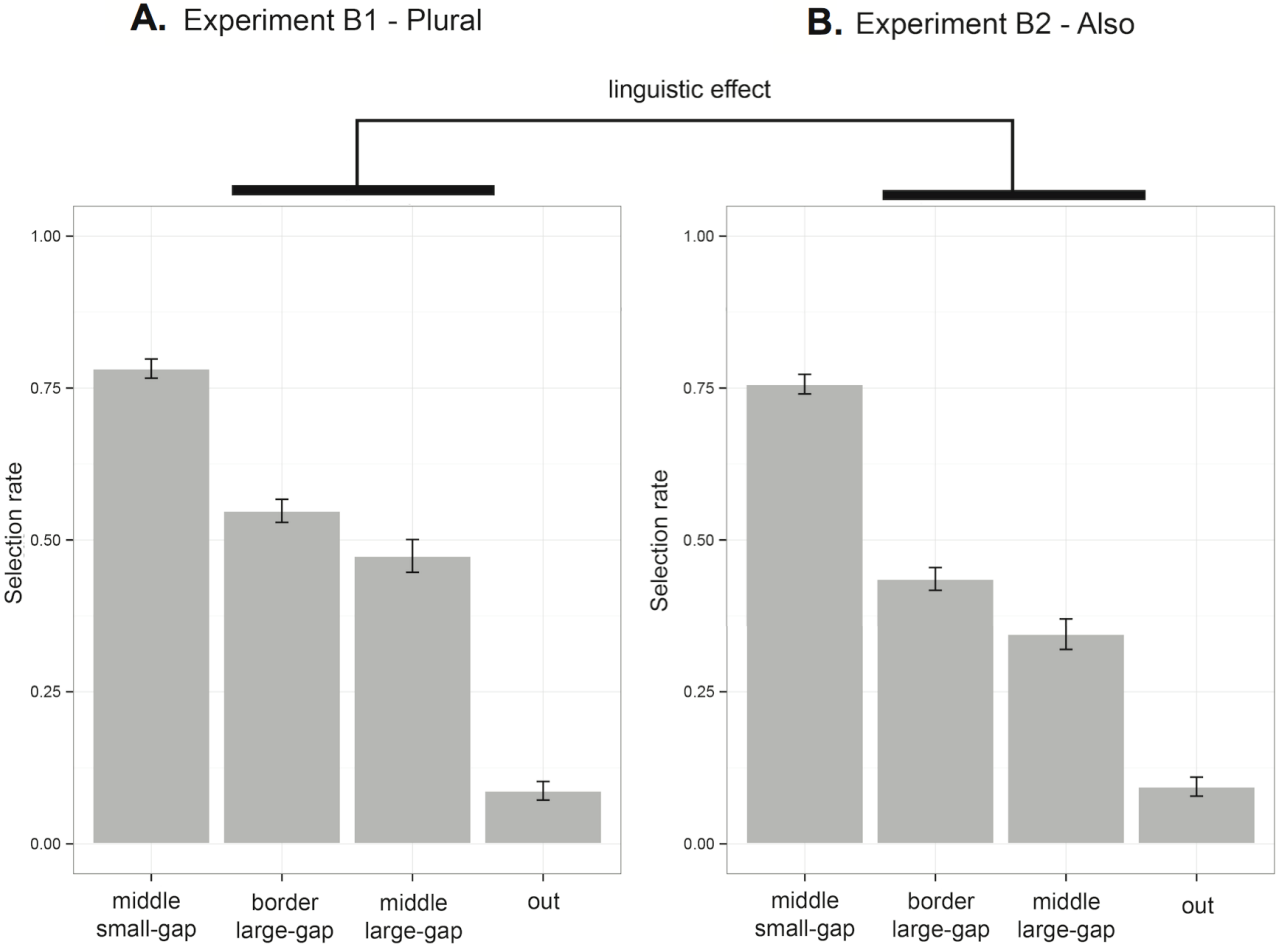
Results of Experiment 2. Proportion of selection of each test item averaged across A) The plural-condition using a linguistic construction discarding the possibility of homophony and B) the also-condition using a linguistic construction more suitable to homophony. Error bars indicate standard errors of the mean.

The results are presented in Fig 6. The pairs (le1, le2) and (le1’, le2’) played symmetric roles, we accordingly collapsed responses for *middle-small-gap* and *middle-small-gap’* and responses for *border-small-gap* and *border-small-gap’* (practically ignoring the prime sign in the report).

First, the results confirm the existence of a gap effect. Participants showed sensitivity to the sampling distribution of the exemplars, in that they selected more *middle-small-gap* than *middle-large-gap* in both the also-condition (β = 2.20, *z* = 11.26, *p* < .001) and the plural-condition (β = 1.70, *z* = 8.59, *p* < .001).

Interestingly, the minimum path (in terms of number of branches) between the learning exemplars le1 and le2 was smaller in Experiment 1A (mean for d(le1, le2) = 3.65), compared both to (le1, le2) and (le1’, le2’) in Experiment 2 (mean for d(le1,le2) = 7.16) (see S1 Supplemental material). Accordingly, we found a cross-experiment gap effect such that *middle-small-gap* was less selected in Experiment 2 (*M =* 0.75) than in Experiment 1A (*M* = 0.95).

Our critical expectation concerned the comparison between linguistic presentations. Test items in the gap between (le1, le2) and (le1’, le2’) were selected more often in the plural-condition than in the also-condition: this was true both for *middle-large-gap* (β = 0.73, *z* = 2.24, *p* < .05) and *border-large-gap* (β = 0.64, *z* = 2.13, *p* < .05), resulting in an interaction effect: the difference between the selection rate of *middle-small-gap* (serving as a baseline) and the combined selection rate of *middle-large-gap* and *border-large-gap* was greater in the plural-condition than in in the also-condition (β = 0.46, *z* = 2.02, *p* < .05; We Helmert coded the predictor Test Item to compare the choice of *middle-small-gap* to the choice of the *middle-large-gap* and *border-large-gap* as a group).

### Discussion

When presented with a plural construction (e.g., “These are blickets”), participants were more likely to associate the word to a category that spans over all the exemplars than when they were presented with a construction compatible with homophony (e.g., “This is a blicket and this is *also* a blicket”). This effect suggests that the gap effect documented in Experiment 1 is the footprint of homophony mapping two words with the same phonological form onto two convex concepts and not of the association of a single word to a discontinuous category.

Certainly, in line with previous results (e.g.,[21], participants were guided in part by similarity: they extended a label more to an object close to the exemplars (*middle-small-gap*) than to an object further away (*middle*-*large-gap*) and they extended the label less to *middle-small-gap* in Experiment 2 than they did in Experiment 1 due to a greater distance between the learning exemplars in Experiment 2 (Note that this is also compatible with the size principle documented by Xu and Tenenbaum (2007) [12]: since the boundaries of the categories defined by the pairs of exemplars (le1, le2) and (le1’, le2’) were less sharp than in Experiment 1A, the correct level of generalization was more uncertain in Experiment 2 than in Experiment 1A.). Yet, such similarity effects cannot explain the main result of Experiment 2 since the linguistic manipulation is realized holding constant similarity relations among learning exemplars and test items. The amount of similarity-driven generalization in participants’ responses could be quantified (see [12] for a model comparison of rule- vs. similarity-based model), but it is sufficient for our purposes to note that it cannot account for the entirety of the present effects, which are driven by linguistic manipulations alone in Experiment 2. Note that while we demonstrate that participants are sensitive to the linguistic constructions in which the words enter, we cannot tell whether the *also* construction alters the results in one direction (towards homophony), or whether the *plural* construction pushes in the opposite direction (against homophony), or both.

One may also ask whether the plural/also effect is linked to the very specific linguistic constructions involved or whether it is merely driven by the visual, two-part presentation that co-varies with these constructions in our experiments. Importantly, a visual effect (i.e., a visual “zeugma”) would make the same point as a more specific linguistic construction effect: all that matters for our argument is that there is room for two tokens of the same phonological form, either because two tokens are indeed present, or simply because the presentation introduces different labeling events.

## General Discussion

We documented two factors that reduce the tendency to map a phonological form onto a single, convex extension, an explicit or implicit assumption about learners in the most explicit implementations of word learning accounts: a) the size of the gap in conceptual space between learning exemplars; b) the presence of an intervening label for entities in that gap. These effects were modulated by linguistic manipulations coherent with the presence/absence of homophony. We submit that when encountering novel words in such situations, learners prefer to postulate homophony, whereby a word form does *not* adhere to a convexity constraint but uncovers two distinct words associated with two convex concepts, thus preserving concept convexity.

In the following we first discuss whether our results depend on the objective definition we adopted for conceptual space. Second, we show how the current study of homophony is relevant to current accounts of word learning broadly, and why other phenomena should be subjected to the same scrutiny. Third, we discuss our findings for a different, albeit similar, phenomenon in the lexicon: polysemy. Finally, our results are based on adult data only and we discuss their relevance for children in the process of learning their native language.

### How to work with concepts

The study of homophony allowed us to examine the existence of a convexity constraint over concepts/words. We started by defining that a concept is convex if its members form a group that share a common set of properties that holds them to be contiguous in conceptual space. The notion of *contiguity* in conceptual space is meaningful as long as the conceptual space is equipped with a metrical structure such as the one we defined throughout this study. One worry however is that there may not be a stable metric between abstract entities across contexts [22], such that two entities can be made arbitrarily similar by changing the dimension under consideration. For instance, one may consider that a Ferrari and a VW Beatle are closer to one another than a Ferrari and a diamond, but this similarity relation may reverse if the context involves paying attention to the value of entities. As a result, one may wonder whether the notion of conceptual similarity may not be too fragile to sustain word learning. This is an important challenge that needs to be looked at carefully, as the current results are incorporated into word learning algorithms. We submit however that in the long run this variability could be controlled if we accept that some “conceptual dimensions” are privileged: they are more stable across contexts, by default, and infants are biased to pay more attention to them (e.g., [23]). For instance, animacy may be a property that is *privileged* in that sense, over say color, to categorize objects. It does not always have to be the case, but on average this will create the basis for a stable set of privileged features to (partly) provide a structure for conceptual space (see also [24] for the notion of *ad hoc* categories and [25], [26] for the idea of concept naturalness).

The next worry then is to decide how one can objectively assess what the actual, “privileged” metric in conceptual space is and derive testable predictions from there. We have seen several responses to this issue: For instance, Xu and Tenenbaum (2007) [12] gathered subjective judgments of similarities, independently from the categorization task. In our studies, we decided on a structure of conceptual space prior to using it for our test. Specifically, our notion of convexity relied on phylogenetic trees and on an arbitrary metric over a multi-dimensional space of visual features. The hope was that there would be a sufficiently good matching between these idealized conceptual spaces and what participants would actually take to be the relations between the relevant entities. Since participants had access to the entities only through visual representations, one may worry that we over-evaluated the chances that perceptual features could determine concepts. Perceptual features as such may not be the determinant of conceptual structure, since concepts may be defined by non-observable properties. Several developmental studies show that, indeed, children prefer to draw inferences based on category membership than inferences based on perceptual appearances (e.g., [27]–[29]). Nevertheless, we use perceptual similarity as a proxy to reflect conceptual structure and follow previous work in that respect (see [12], [30]–[33]). Interestingly, we note that young children may also use such a proxy in their earliest word meaning inferences [9], [34].

The current inquiry was based on the hope that conceptual space could be approximately circumscribed by objective or scientifically based properties (e.g., phylogenetic trees). There surely has to be *some* correlation between such an objectively based categorization and actual, subjective categorization (e.g., [35]). Most importantly, the fact that our results come out the right way suggests *a posteriori* that our simplifying hypotheses are acceptable to a sufficient degree: our results could not be obtained if our assumptions to approximate the underlying conceptual structure were inappropriate.

### Challenges for accounts of word learning

The above discussion only refers to concepts, not to words. A natural assumption is that one word would map onto one concept, but it does not have to be so. For instance, a word could map onto a set of concepts, as if there was a word meaning dog or bone (where dog and bone here are supposed to be disjoint concepts). Our study of homophones shows that this does not happen. Instead, when a word could potentially have such a disjunctive, discontinuous meaning, a homophone is created.

As we described in the introduction, convexity holds for words and not for word forms. This distinction was not and could not be investigated through previous experimental work since word learning studies focused on non-homophonous cases, for which word forms and words are confounded (e.g., [7], [8]). As a result, nothing prevented the computational implementation of these accounts to hardwire the efficient convexity constraint on word forms as a systemic property of the learning mechanism, but the current results force us to reflect on the status and consequence of this assumption. To date, word learning models capitalize on a convexity constrain on word forms, whereby word forms map onto a single meaning that ought to be convex in conceptual space, as this is the most natural version to implement for a learner who is only exposed to word forms. One possibility is that the convexity constrain on word forms is a mere heuristic, that would provide the right outcome in most word learning circumstances, where word forms and words are equivalent. Yet, the existence of homophony suggests that learners must be able to detect cues that would help them depart from their default over-simplification, and relax their word form convexity heuristic into a concept convexity constraint. Importantly, if the learner deploys such a heuristic, the system must be able to decide when to trigger and when to silence it and we documented the empirical cues under which this switch may happen. But this leaves open a fundamental question about when the system is capable of such a sophistication: should we assume that words and the abstract notion of word forms are both primitive of the learning system? This would be an interesting and striking innateness type of claim, which should be assessed carefully. We saw that by specializing on a subset of cases (neglecting homophony), current word learning accounts missed to address that discussion (and actually ended up banning homophony from the system entirely).

Let us illustrate with another example showing that the assumptions behind word learning algorithms should be scrutinized with care when they focus their attention on a subclass of phenomena. Xu and Tenenbaum (2007) [12] propose a rational use of co-occurrences of words and objects to learn the meaning of words. But this crucially applies to content words only. Function words, however, occur in all sorts of contexts and may co-occur with all possible objects, in principle. Hence, without further assumptions, the model predicts that words like “the” or “and” mean the same as “thing” or “stuff”, which also co-occur with any kind of object. Arguably, learners deploy a different strategy to learn content words and function words (see relatedly [36] for the use of a different strategy for learning numerical concepts). But how does the learning system separate hypothesis spaces and learning algorithms for content words and function words? Ideally, one would propose a device, say a sorting algorithm, which capitalizes on differences between these words to orient function words and content words to the right learning algorithms for principled reasons (see, e.g., [37] for *empirical* facts about the relative frequency of function and content words that could be the input of such a sorting mechanism). But until such a mechanism is exhibited, the separation of word learning into sub-algorithms which selectively apply to different classes of words comes at the cost of presupposing that learners innately expect the distinction between these classes (i.e. that languages contain both function words and content words, or that languages contain homophones and non-homophones to go back to our central case).

In sum, current word learning accounts break the learning problem into manageable pieces of the puzzle, studying object labels, ambiguous words, functions words or numerical concepts separately. A reconciliation of these pieces into a single solution may be technically easy; one could say that the system “expects” these differences. But it has rich consequences because in the absence of a more complete picture, it amounts to postulating that subtle and quite specific phenomena such as the distinction between function words and content words or the existence of homophony have an innate basis.

### Homophony and Polysemy

There is a rich literature distinguishing homophony from polysemy. In short, polysemy is taken to be a form of motivated homophony, by which a word has two related meanings, with possibly systematic variation (e.g., [38]–[41]). For example, an object may take the same label as the artist who made it (e.g., Picasso / a Picasso) and this process is productive (e.g., “The museum owns a *Mandela*” would lead one to infer that Nelson Mandela was a painter). The current literature assumes that such productivity is caused by the presence of generative lexical or conceptual structures that allow meanings to be generated from the context (e.g., [40], [41]). As a result, the representation of polysemous words, for which different meanings are generated from a single entry, is different from the representation of homophones, for which different meanings are stored separately (e.g., [42]–[44]).

We showed that a word is more likely to yield homophony if it labels different portions of the conceptual space. In its simplest version, this would entail that learners would postulate homophony when facing polysemy, as the meanings of a polysemous word also label distant locations in conceptual space (e.g., Picasso, a human man and its painting, an artifact). Yet we know that this is not the case: Children as young as 4 years of age are able to distinguish polysemy from homophony ([45], [46]). Thus, any account of word learning must be able to explain how learners distinguish between homophony and polysemy, surely, capitalizing on the systematic link that exists between the different meanings of a polysemous word, a systematic link which exists even if the meanings are separated in conceptual space.

Depending on how we assume that polysemy is represented, the challenge may take a different form. For the sake of concreteness, assume that polysemy is underlined by Meaning Shift operations, MS, which transform the meaning of a word in systematic ways, (e.g., in the Picasso/Mandela example above, the relevant MS could be akin to “a piece of art made by…”) (see however [47], [48] for the hypothesis that the interpretation of polysemic words depends on pragmatic, gricean reasoning rather than on rule-like processes that generate an extended meaning from an existing one). Hence, polysemy does not involve a different *word* learning process, but the discovery of yet another layer of representation, that of the MS operations, which applies to words. Hence, *Picasso* really is a simple word, the name of an artist, but it can be interpreted as MS(*Picasso*). In practice then, if a word seems to apply to a non-convex set of examples, a learner would have three choices: (i) opt out from ambiguity altogether and postulate a broad meaning for the word, (ii) postulate ambiguity: assume that the word form corresponds to two distinct words, (iii) postulate polysemy: choose one of the meaning as primitive and postulate an MS operation to account for the rest of the examples. We have shown conditions under which learners prefer (ii) over (i), the conditions under which learners prefer (iii) remain to be studied.

### Early language acquisition

Through the study of homophones, our studies uncover several factors that play an important role in revealing the existing constraints on how words associate with concepts in general. An important open question is whether these factors influence word learning during the earliest stages of word acquisition (see Dautriche, Chemla & Christophe, *in press*, for initial investigations with 4 year olds). While studying adults may inform us about the general strategies involved in word learning [49], children have different cognitive resources and biases and may consequently use different strategies. We discuss four factors that could lead to the emergence of homophony in children:

1. *Concept convexity*. Adults refrain from associating a label to a broad concept when positive evidence is missing for a large gap within the concept. The observation that a label applies to a discontinuous extension triggers the formation of novel word representations that are compatible with concept convexity. Do children also expect words to refer to coherent and convex concepts and, if so, what representation do they adopt when the convexity constraint over word forms is not met? [50] offer a relevant study in which they presented 10-month-old infants with exemplars of a word forming a gap in conceptual space: the presence of a similar label was enough for children to extend the label to all intervening items in that gap. Yet, they only tested rather small gaps, which may very well be before the breaking point of the convexity constraint over word forms.
2. *Sampling effect*. [12] document a “size principle” according to which the sharpness of a concept is a function of the number of learning exemplars, for both children and adults. We showed an effect of the *distribution* of the learning exemplars in conceptual space: observing exemplars clustered at two distant positions in the hypothesis space boosted the likelihood that the exemplars were sampled from two independent categories. Children are sensitive to the size principle; they may also show sensitivity to such a “distribution principle”, a possibility that we explored elsewhere [51].
3. *The structure of the semantic lexicon*. When confronted with a new word, adults consider the existence of *other* (potentially unknown) words. Specifically, they generalize a word A less to a new object if this new object comes in the vicinity of a concept labeled by a word B. This demonstrates that learners have expectations about the structure of the semantic lexicon as a whole and priors about how words may share the conceptual space. This new kind of evidence against individual word-by-word learning is coherent with simpler, so-called “mutual exclusivity effects” [15], according to which a new word should not occupy the same conceptual space as a known word. Interestingly, this effect has to be modulated by other factors, since some words surely overlap in conceptual space (e.g., compare *cat* and *animal*). To our knowledge, priors over the whole lexicon are missing from current word learning computational models—and their implementation raises immediate challenges.
4. *Linguistic factors*: Adults’ generalization was modulated by the linguistic construction in which words were presented. While we used linguistic constructions as a linguistic test for homophony, these constructions may also be used to discover homophony (noting that homophones never appear in plural constructions but may appear in some more appropriate constructions such as the *also* construction we documented). Whether children are able to pick up on this is an empirical question, both because they may not be sensitive to these linguistic factors (effectively this would otherwise be a case of linguistic bootstrapping of homophony) or because the relevant facts may be too sparse in their input, e.g., if homophones cover distant concepts, it is unlikely that these two concepts will be mentioned within the same learning situation.

### Summary

In this work, we showed that a word is more likely to yield homophony if: (a) it is learnt from exemplars leaving an important gap between them (in conceptual space), (b) this gap in conceptual space is occupied by other words. We submit that encountering novel words in such situations may trigger forms of word representations which comply with concept convexity. More generally, we argue that incorporating homophony and other challenging word learning phenomena into current word learning accounts, will provide a better understanding of learners’ implicit knowledge and assumptions about how word forms map onto meanings.

## Supporting Information

S1 Supplemental Material (DOCX)

## References

[1] Frege G., “Ueber Begriff und Gegenstand,” *Vierteljahr*. *Fuer Wiss*. *Philos.*, vol. 16, pp. 192–205, 1892.

[2] Bloom P., “Précis of How children learn the meanings of words,” *Behav*. *Brain Sci.*, vol. 24, no. 06, pp. 1095–1103, 2001. 12412326 10.1017/s0140525x01000139

[3] Goodman N., *Fact*, *fiction*, *and forecast*. Harvard University Press, 1955.

[4] Keil F. C., *Concepts*, *kinds*, *and cognitive development*. MIT Press, 1989.

[5] Markman E. M., *Categorization and naming in children*: *Problems of induction*. Mit Press, 1989.

[6] Mitchell T. M., *The need for biases in learning generalizations*. Department of Computer Science, Laboratory for Computer Science Research, Rutgers Univ., 1980.

[7] Markman E. M. and Hutchinson J. E., “Children’s sensitivity to constraints on word meaning: Taxonomic versus thematic relations,” *Cognit*. *Psychol.*, vol. 16, no. 1, pp. 1–27, 1984.

[8] Waxman S. and Gelman R., “Preschoolers’ use of superordinate relations in classification and language,” *Cogn*. *Dev.*, vol. 1, no. 2, pp. 139–156, 1986.

[9] Landau B., Smith L. B., and Jones S. S., “The importance of shape in early lexical learning,” *Cogn*. *Dev.*, vol. 3, no. 3, pp. 299–321, 1988.

[10] Gardenfors P., “Conceptual spaces as a framework for knowledge representation,” *Mind Matter*, vol. 2, no. 2, pp. 9–27, 2004.

[11] Murphy G. L. and Medin D. L., “The role of theories in conceptual coherence.,” *Psychol*. *Rev.*, vol. 92, no. 3, p. 289, 1985. 4023146

[12] Xu F. and Tenenbaum J. B., “Word learning as Bayesian inference.,” *Psychol*. *Rev.*, vol. 114, no. 2, pp. 245–272, 2007. 17500627 10.1037/0033-295X.114.2.245

[13] Siskind J. M., “A computational study of cross-situational techniques for learning word-to-meaning mappings,” *Cognition*, vol. 61, no. 1, pp. 39–91, 1996. 8990968 10.1016/s0010-0277(96)00728-7

[14] Regier T., “The emergence of words: Attentional learning in form and meaning,” *Cogn*. *Sci.*, vol. 29, no. 6, pp. 819–865, 2005. 21702796 10.1207/s15516709cog0000_31

[15] Markman E. M. and Wachtel G. F., “Children’s use of mutual exclusivity to constrain the meanings of words,” *Cognit*. *Psychol.*, vol. 20, no. 2, pp. 121–157, 1988. 3365937 10.1016/0010-0285(88)90017-5

[16] Jaeger T. F., “Categorical data analysis: Away from ANOVAs (transformation or not) and towards logit mixed models,” *J*. *Mem*. *Lang.*, vol. 59, no. 4, pp. 434–446, Nov. 2008. 19884961 10.1016/j.jml.2007.11.007 PMC2613284

[17] Bates D. and Sarkar D., Ime4 library. Accessed, 2004.

[18] Cruse D. A., *Lexical semantics*. Cambridge University Press, 1986.

[19] Zwicky A. and Sadock J., “Ambiguity tests and how to fail them,” *Syntax Semant.*, vol. 4, no. 1, pp. 1–36, 1975.

[20] Geeraerts D., “Vagueness’s puzzles, polysemy’s vagaries,” *Cogn*. *Linguist*. *Incl*. *Cogn*. *Linguist*. *Bibliogr.*, vol. 4, no. 3, pp. 223–272, 1993.

[21] Goldstone R. L., “Arguments for the Insufficiency of Similarity for Grounding Categorization,” 1994.

[22] Tversky A., “Similarity features,” *Psychol*. *Rev.*, vol. 84, pp. 327–352, 1977.

[23] Poulin-Dubois D., Lepage A., and Ferland D., “Infants’ concept of animacy,” *Cogn*. *Dev.*, vol. 11, no. 1, pp. 19–36, 1996.

[24] Barsalou L. W., “Ad hoc categories,” *Mem*. *Cognit.*, vol. 11, no. 3, pp. 211–227, 1983. 10.3758/bf03196968 6621337

[25] Keil F. C., “Constraints on knowledge and cognitive development.,” *Psychol*. *Rev.*, vol. 88, no. 3, pp. 197–227, 1981.

[26] Osherson D. N., “Three conditions on conceptual naturalness,” *Cognition*, vol. 6, no. 4, pp. 263–289, 1978.

[27] Gelman S. A. and Coley J. D., “The importance of knowing a dodo is a bird: Categories and inferences in 2-year-old children.,” *Dev*. *Psychol.*, vol. 26, no. 5, p. 796, 1990.

[28] Gelman S. A. and Markman E. M., “Young children’s inductions from natural kinds: The role of categories and appearances,” *Child Dev.*, pp. 1532–1541, 1987 3691200

[29] Graham S. A., Kilbreath C. S., and Welder A. N., “Thirteen-Month-Olds Rely on Shared Labels and Shape Similarity for Inductive Inferences,” *Child Dev.*, vol. 75, no. 2, pp. 409–427, 2004. 15056196 10.1111/j.1467-8624.2004.00683.x

[30] Medin D. L. and Schaffer M. M., “Context theory of classification learning.,” *Psychol*. *Rev.*, vol. 85, no. 3, p. 207, 1978.

[31] Nosofsky R. M., “Attention, similarity, and the identification–categorization relationship.,” *J*. *Exp*. *Psychol*. *Gen.*, vol. 115, no. 1, p. 39, 1986. 2937873 10.1037//0096-3445.115.1.39

[32] Shepard R. N., “Attention and the metric structure of the stimulus space,” *J*. *Math*. *Psychol.*, vol. 1, no. 1, pp. 54–87, 1964.

[33] Smith E. E. and Medin D. L., *Categories and concepts*. Harvard University Press Cambridge, MA, 1981.

[34] Graham S. A. and Poulin-Dubois D., “Infants’ reliance on shape to generalize novel labels to animate and inanimate objects,” *J*. *Child Lang.*, vol. 26, no. 02, pp. 295–320, 1999. 11706467 10.1017/s0305000999003815

[35] Atran S., “Folk biology and the anthropology of science: Cognitive universals and cultural particulars,” *Behav*. *Brain Sci.*, vol. 21, no. 04, pp. 547–569, 1998. 10097021 10.1017/s0140525x98001277

[36] Piantadosi S. T., Tenenbaum J. B., and Goodman N. D., “Bootstrapping in a language of thought: A formal model of numerical concept learning,” *Cognition*, vol. 123, no. 2, pp. 199–217, 5 2012. 22284806 10.1016/j.cognition.2011.11.005

[37] Hochmann J.-R., “Word frequency, function words and the second gavagai problem,” *Cognition*, vol. 128, no. 1, pp. 13–25, Jul. 2013. 23557599 10.1016/j.cognition.2013.02.014

[38] Apresjan J. D., “Regular polysemy,” *Linguistics*, vol. 12, no. 142, pp. 5–32, 1974.

[39] Bréal M., *Essai de sémantique*:*(science des significations)*. Hachette, 1904.

[40] Copestake A. and Briscoe T., “Semi-productive polysemy and sense extension,” *J*. *Semant.*, vol. 12, no. 1, pp. 15–67, 1995.

[41] Pustejovsky J., “The generative lexicon,” *Comput*. *Linguist.*, vol. 17, no. 4, pp. 409–441, 1991.

[42] Caramazza A. and Grober E., “Polysemy and the structure of the subjective lexicon,” *Georget*. *Univ*. *Roundtable Lang*. *Linguist*. *Semant*. *Theory Appl.*, pp. 181–206, 1976.

[43] Rabagliati H. and Snedeker J., “The Truth About Chickens and Bats: Ambiguity Avoidance Distinguishes Types of Polysemy,” *Psychol*. *Sci.*, 5 2013. 10.1177/0956797612472205 23722978

[44] Seidenberg M. S., Tanenhaus M. K., Leiman J. M., and Bienkowski M., “Automatic access of the meanings of ambiguous words in context: Some limitations of knowledge-based processing,” *Cognit*. *Psychol.*, vol. 14, no. 4, pp. 489–537, 1982.

[45] Srinivasan M. and Snedeker J., “Judging a book by its cover and its contents: The representation of polysemous and homophonous meanings in four-year-old children,” *Cognit*. *Psychol.*, vol. 62, no. 4, pp. 245–272, Jun. 2011. 21530473 10.1016/j.cogpsych.2011.03.002

[46] Srinivasan M. and Snedeker J., “Polysemy and the Taxonomic Constraint: Children’s Representation of Words that Label Multiple Kinds,” *Lang*. *Learn*. *Dev.*, vol. 10, no. 2, pp. 97–128, 2014.

[47] Nunberg G., “The non-uniqueness of semantic solutions: Polysemy,” *Linguist*. *Philos.*, vol. 3, no. 2, pp. 143–184, 1979.

[48] Nunberg G., “Transfers of meaning,” *J*. *Semant.*, vol. 12, no. 2, pp. 109–132, 1995.

[49] Markson L. and Bloom P., “Evidence against a dedicated system for word learning in children,” *Nature*, vol. 385, no. 6619, pp. 813–815, 1997. 9039912 10.1038/385813a0

[50] Plunkett K., Hu J.-F., and Cohen L. B., “Labels can override perceptual categories in early infancy,” *Cognition*, vol. 106, no. 2, pp. 665–681, Feb. 2008. 17512515 10.1016/j.cognition.2007.04.003

[51] Dautriche I., Chemla E., and Christophe A., “Word Learning: Homophony and the Distribution of Learning Exemplars,” *Lang*. *Learn*. *Dev.*, vol. 12, no. 3, pp. 231–251, 2016.

