# Supplementary material for: What Homophones Say about Words
Source: PLoS One. 2016 Sep 1;11(9):e0162176. doi: 10.1371/journal.pone.0162176 (PMC5008697; doi:10.1371/journal.pone.0162176)
Supplement: S1 Supplemental Material — (DOCX) [file pone.0162176.s001.docx]

**Supplemental Material**

**Experiment 1A: Natural stimuli**

The experimental materials consisted of 146 objects. The objects were distributed across three biological kingdoms: animals (100), plants (30) and fungi (16). We did not include species for which there is a clear difference between scientific and subjective popular taxonomy (e.g., sea mammals: counter-intuitively, dolphins are closer to elephants than to sharks). For each object, we selected 3 color photographs (EOL, <http://eol.org>) showing it in its natural background. We used the taxonomy database from NCBI (http://www.ncbi.nlm.nih.gov) to obtain an objective measure of similarity between the objects. Each trial involved learning exemplars (le_1_, le_2_, le_3_, le_X_) and test items (*middle-small-gap, border-large-gap, middle-large-gap, out*) selected according to the schema in Figure 1.

We define the distance d between two items A and B as the length (in number of branches) of the minimum path that connects them. We also define S(A, B) the minimal, well-formed subtree containing A and B. Not that we did not count as well-formed subtrees those for which the parent of its root node was not more than 20% older. This was done to account "category distinctiveness" from Xu & Tenenbaum (2007) (exemplified by the idea that reptiles may be a well-formed category but not "reptiles but turtles"). The age of the common ancestors were obtained from <http://www.timetree.org/>.

*Test trials.* The learning exemplars le_1_, le_2_ and le_3_ were chosen such that d(le_1_, le_2_) ≤ *6* and d(le_2_, le_3_) was between 3 and 5 times larger than d(le_1_, le_2_)*.*

The test items (*middle-small-gap, border-large-gap, middle-large-gap, out)* and the intervening learning exemplar (le_X_) were chosen such that:

1) *middle-small-gap* belong to the smallest subtree containing le_1_ and le_2_, S(le_1_, le_2_). And d(*middle-small-gap*, le_1_) < d(*middle-small-gap*, le_2_).

2) *border-large-gap* was *not* in S(le_1_, le_2_) but it was among the closest elements to S(le_1_, le_2_), i.e. an element that *must* be added to S(le_1_, le_2_) to obtain the next bigger well-formed subtree.

3) *imiddle-large-gap* was not in S(le_1_*, border-large-gap)* and le_3_ was not in S(le_1_*, middle-large-gap)*.

The distances were constrained such that d(le_1_*, middle-large-gap)* and d(le_3_*, middle-large-gap) <* d(le_1_, le_3_)*.*

4) *out* was not in S(le_1_, le_2_, le_3_) and d(*out*, *middle-large-gap*) > d(*out,* le_1_).

5) le_X_ was not in S(le_1_, *border-large-gap*) but it was in S(le_1_*, middle-large-gap*) such that d(le_1_, le_X_) *>* d(le_1_, *border-large-gap*) and d(le_1_*, middle-large-gap*) > d(*middle-large-gap*, le_X_).

*Filler trials.* The three learning examples le_1_, le_2_, le_3_ were evenly distributed (average distance = 5).

1. In the 6 attractive fillers, at least 3 test items were in S(le_1_, le_2_, le_3_); the remaining 1 or 2 test items were maximally distant from learning exemplars: there was no well-formed subtree containing the test items and the learning exemplars except the whole tree.

Three of these attractive filler items included a fourth learning example with another label, as in the Intervention trials. This item was maximally distant to the learning exemplars (in the sense explained above).

1. In the 3 repulsive fillers, 0 or 1 test item was in S(le_1_, le_2_, le_3_); the remaining 3 or 4 items were maximally distant from these learning exemplars.

These constraints allowed for 4037 and 6353 possible configurations for Experiment 1A and 1B respectively.

**Experiment 1B: Artificial stimuli**

The stimuli were arrangements of geometric shapes, as exemplified in Figure 3. They were created by combining five parameters with four possible levels each: core pattern (1 circle, 4 overlapping circles, 4 tangent circles, 4 independent circles), core pattern occurrences (1 to 4), size of the core pattern, number of radial lines (1 to 4) and number of bumps in the radial lines (0, 1, 2, 8). The total number of possible items was thus 4^5^=1024.

The distance between two such items was defined as the so-called Manhattan distance in their multi-dimensional space, i.e. as the sum of the distances within each dimension. As an example of application, the maximal distance between two items was 15 = 3(max distance within a given dimension) x 5(number of dimensions).

*Test trials.* The 3 learning exemplars, le_1_, le_2_, le_3_ were chosen such that le_1_ and le_2_ varied along a single dimension D_1,2_ and d(le_1_, le_3_) = 4 x d(le_1_, le_2_), following the 4:1 ratio for the natural stimuli. The 4 test items were such that:

1) *middle-small-gap* is equidistant to le_1_ and le_2_ along this dimension D_1,2_.

2) *border-small-gap* shares all but one of the features common to le_1_, le_2_ and *middle-small-gap*.

3) *middle-large-gap* shares all features common to le_1_ and le_3_*.* And d(*middle-large-gap*, le_1_) = d(*middle-large-gap*, le_3_).

4) *out* does not have any common feature with le_2_ and le_3_. And d(le_2_, *out*) = d(le_3_, *out*).

*Filler trials.* The 3 learning exemplars were chosen such that le_1_ and le_2_ varied along a single dimension and d(le_1_, le_2_)=1 and d(le_2_, le_3_)=4.

1. In attractive filler trials, 3 or 4 test items were “attractive”, i.e. they were designed to attract selection: they shared all the features common to le_1_*,* le_2_ and le_3_. The fourth test item was “repulsive”: it did not have any feature in common with the learning exemplars.

Half of these filler items included an additional learning exemplar labeled with another word, this learning exemplar was somehow irrelevant in that it shared no feature with any of the other learning exemplars.

1. In repulsive filler trials, 3 test items were repulsive and 1 was attractive.

**Experiment 2**

The items were selected following the criteria described in Experiment 1A except that (a) 6 ≤ d(le_1_,le_2_) ≤ 12 and similarly 6 ≤ d(le_1_’,le_2_’) ≤ 12, (compare to d(le_1_, le_2_) ≤ 6 in Experiment 1A) and (b) the distances between le_1_ or le_2_ and le_1_’ or le_2_’ were twice as large as the internal distances in the pairs. To compare with Experiment 1A: the learning exemplars span a subtree of the same size, the gap was narrower.

The total number of possible configurations satisfying these constraints was 145.
